# Integrative multi-platform meta-analysis of gene expression profiles in pancreatic ductal adenocarcinoma patients for identifying novel diagnostic biomarkers

**DOI:** 10.1371/journal.pone.0194844

**Published:** 2018-04-04

**Authors:** Antonio Irigoyen, Cristina Jimenez-Luna, Manuel Benavides, Octavio Caba, Javier Gallego, Francisco Manuel Ortuño, Carmen Guillen-Ponce, Ignacio Rojas, Enrique Aranda, Carolina Torres, Jose Prados

**Affiliations:** 1 Department of Medical Oncology, Virgen de la Salud Hospital, Toledo, Spain; 2 Institute of Biopathology and Regenerative Medicine (IBIMER), Center of Biomedical Research (CIBM), University of Granada, Granada, Spain; 3 Department of Medical Oncology, Virgen de la Victoria Hospital, Malaga, Spain; 4 Department of Health Sciences, University of Jaen, Jaen, Spain; 5 Department of Medical Oncology, University General Hospital of Elche, Alicante, Spain; 6 Department of Computer Architecture and Computer Technology, Research Center for Information and Communications Technologies, University of Granada, Granada, Spain; 7 Department of Medical Oncology, Hospital Ramón y Cajal Hospital, Madrid, Spain; 8 Maimonides Institute of Biomedical Research (IMIBIC), Reina Sofia Hospital, University of Cordoba, Cordoba, Spain; 9 Department of Biochemistry and Molecular Biology I, Faculty of Sciences, University of Granada, Granada, Spain; University of Nebraska Medical Center, UNITED STATES

## Abstract

Applying differentially expressed genes (DEGs) to identify feasible biomarkers in diseases can be a hard task when working with heterogeneous datasets. Expression data are strongly influenced by technology, sample preparation processes, and/or labeling methods. The proliferation of different microarray platforms for measuring gene expression increases the need to develop models able to compare their results, especially when different technologies can lead to signal values that vary greatly. Integrative meta-analysis can significantly improve the reliability and robustness of DEG detection. The objective of this work was to develop an integrative approach for identifying potential cancer biomarkers by integrating gene expression data from two different platforms. Pancreatic ductal adenocarcinoma (PDAC), where there is an urgent need to find new biomarkers due its late diagnosis, is an ideal candidate for testing this technology. Expression data from two different datasets, namely Affymetrix and Illumina (18 and 36 PDAC patients, respectively), as well as from 18 healthy controls, was used for this study. A meta-analysis based on an empirical Bayesian methodology (ComBat) was then proposed to integrate these datasets. DEGs were finally identified from the integrated data by using the statistical programming language R. After our integrative meta-analysis, 5 genes were commonly identified within the individual analyses of the independent datasets. Also, 28 novel genes that were not reported by the individual analyses (‘gained’ genes) were also discovered. Several of these gained genes have been already related to other gastroenterological tumors. The proposed integrative meta-analysis has revealed novel DEGs that may play an important role in PDAC and could be potential biomarkers for diagnosing the disease.

## Introduction

Pancreatic ductal adenocarcinoma (PDAC), the most common type of pancreatic cancer (PC), is the fourth leading cause of cancer death in Western countries, with a 5-year survival rate of about 4% and a median survival rate of less than 6 months [1]. At the time of diagnosis, 80% of patients with PDAC are found to have unresectable locally advanced or metastatic disease [2]. The absence of reliable biomarkers for population screening is one of the most important limitations in the management of this malignancy [3].

Currently, the only biomarker in routine clinical use for PDAC is the carbohydrate antigen 19–9 (CA19-9) [4]. However, recent studies found this biomarker to be an unreliable diagnostic tool due to its limited sensitivity (~80%) and specificity (80–90%) [5]. Furthermore, elevated levels of CA19–9 may also appear in pancreatitis [6], benign diseases of the hepatobiliary system [7] and other malignancies of the gastrointestinal tract [8].

Microarray techniques have become a useful tool for determining gene expression profiles in cancer, allowing the discovery of possible tumor biomarkers [9]. However, sometimes biopsy from tumoral tissues can be complex and present complications. In this context, peripheral blood mononuclear cells (PBMCs) constitute an alternative, non-invasive source for finding tumor biomarkers [10,11]. These cells suffer modifications in their gene expression profile when in contact with the tumor microenvironment [12], and may therefore be used as an accessible source of cancer biomarkers.

Additionally, the so-called meta-analysis techniques have been increasingly employed to integrate data from different microarray platforms, making this technology more consistent and powerful. These meta-analyses are especially useful for combining several datasets related to the same disease when they are limited in size, therefore improving their statistical power [13]. Meta-analyses have recently been applied to identify DEGs in several tumor studies, including in breast [14,15], ovarian [16], prostate [17] and pancreatic cancers [18]. One of the main challenges in a meta-analysis is to adequately integrate datasets obtained using different platforms in order to make them comparable. Various methods have been developed to normalize datasets and provide reliable integration, removing batch effects and making cross-platform corrections, such as Distance Weighted Discrimination (DWD) [19], empirical Bayes methods (ComBat) [20], and cross-platform normalization (XPN) [21]. In this sense, ComBat and XPN have been proven to outperform DWD in term of minimizing inter-platform variance [13].

In this study, an integrated meta-analysis of two gene expression datasets from PDAC data was proposed for identifying DEGs in patients. The datasets were collected from two different microarray platforms, namely Affymetrix and Illumina. The expression data was integrated using an empirical Bayes method (ComBat) to avoid bias between the platforms.

## Materials and methods

### Study population

All clinical investigations were conducted according to the principles expressed in the Declaration of Helsinki. All participants gave written informed consent to participate before their enrolment in the study. The study was approved by the respective Ethics Committee at the Hospital Universitario Puerta del Mar, Hospital Germans Trias i Pujol, Complejo Hospitalario de Navarra, Hospital Reina Sofia, Hospital General de Valencia, Hospital Sant Pau, Hospital Virgen de la Salud, Hospital Parc Taulí, Hospital Universitario Ramón y Cajal, Hospital Carlos Haya, Hospital Universitario Marques de Valdecilla, Hospital General de Elche, Hospital Son Llatzer, Hospital Universitario de Donostia, and Hospital Virgen de las Nieves.

The 54 patients with unresectable PDAC recruited in this study were divided into two independent cohorts. Samples from cohort 1, selected from our previous study [22], include 18 patients with PDAC recruited from January 2009 to July 2012 at the Virgen de las Nieves University Hospital in Granada. Cohort 2 was also independent and included 36 new patients with PDAC, from a phase 2 randomized trial, recruited from March 2012 to February 2013 from 15 different hospitals mediated by the Spanish cooperative group for gastrointestinal tumor therapy (TTD). The diagnosis of PDAC was based on clinical evaluation and imaging studies, which were histologically confirmed by surgery or imaging-guided biopsy. The same enrolment criteria were applied to both cohorts. Finally, 18 gender-, age-, and habit- matched healthy controls were included. The study was approved by the Ethics Committee of the different hospitals, and all clinical investigations were conducted according to the principles expressed in the Declaration of Helsinki. Written informed consent was obtained from all patients and controls before their enrolment in the study.

### Blood collection and isolation of total RNA from PBMCs

Prior to any chemotherapy regimen, peripheral blood samples (12 ml) from all patients and healthy controls were collected in PAXgene Blood RNA Tubes (PreAnalytix) and stored at room temperature for 24 hours, to achieve complete lysis of the blood cells and immediate and persistent RNA stabilization. The RNA from PBMCs was isolated using the PAXgene Blood RNA Kit (PreAnalytix) according to the manufacturer's instructions. The final concentration of purified RNA was quantified by absorbance at 260 nm in a NanoDrop 2000c spectrophotometer (Thermo Scientific). The quality was determined using the 2100 Bioanalyzer (Agilent Technologies). All samples presented an RNA integrity number (RIN) >7.0 and a 28S:18S rRNA ratio >1.0.

### cDNA microarray analysis

Whole genome cDNA microarray hybridization of samples was performed using two different platforms to identify potential PDAC markers. Affymetrix microarray-based gene expression profiling was carried out on the samples from the patients included in Cohort 1 and 18 healthy controls, using GeneChip® Human Gene ST 1.0 Arrays (Affymetrix Inc.) according to the recommended protocol. Briefly, l μg of high-quality total RNA was used to synthesize double-stranded cDNA, and biotin-tagged cRNA was produced. This cRNA was recovered, purified and then hybridized to the chips overnight at 45°C. After being washed and stained, the arrays were scanned with a GeneChip Scanner 3000 7G (Affymetrix Inc.) following the manufacturer’s protocol.

The gene expression levels of multi-plat were measured using the HumanHT-12 v4 Expression BeadChip (Illumina Inc.). In addition, the expression data for the same 18 healthy controls were recalculated using Illumina technology. Both Affymetrix and Illumina expression values from healthy controls were considered for the integrative meta-analysis. Briefly, 1 μg of high-quality total RNA isolated using the Illumina TotalPrep RNA Amplification Kit (Ambion) was amplified. Then, it was reverse transcripted into first and second strand cDNA, and biotin labeled cRNA were generated following the manufacturer’s instructions. This labeled cRNA was hybridized overnight to the arrays. The beadchips were washed, stained with dye-labeled streptavidin, and scanned with an Illumina IScan to measure the intensity. The raw data images were analyzed with Illumina Genome Studio software, which generated an average probe intensity for each sample.

Data deposition: the data from both microarrays reported in this paper were deposited in the Gene Expression Omnibus (GEO) database (http://www.ncbi.nlm.nih.gov/geo) with accession numbers GSE49641 and GSE74629 for the Affymetrix and Illumina platforms, respectively.

### Microarray data processing and integrative meta-analysis

**Fig 1:**
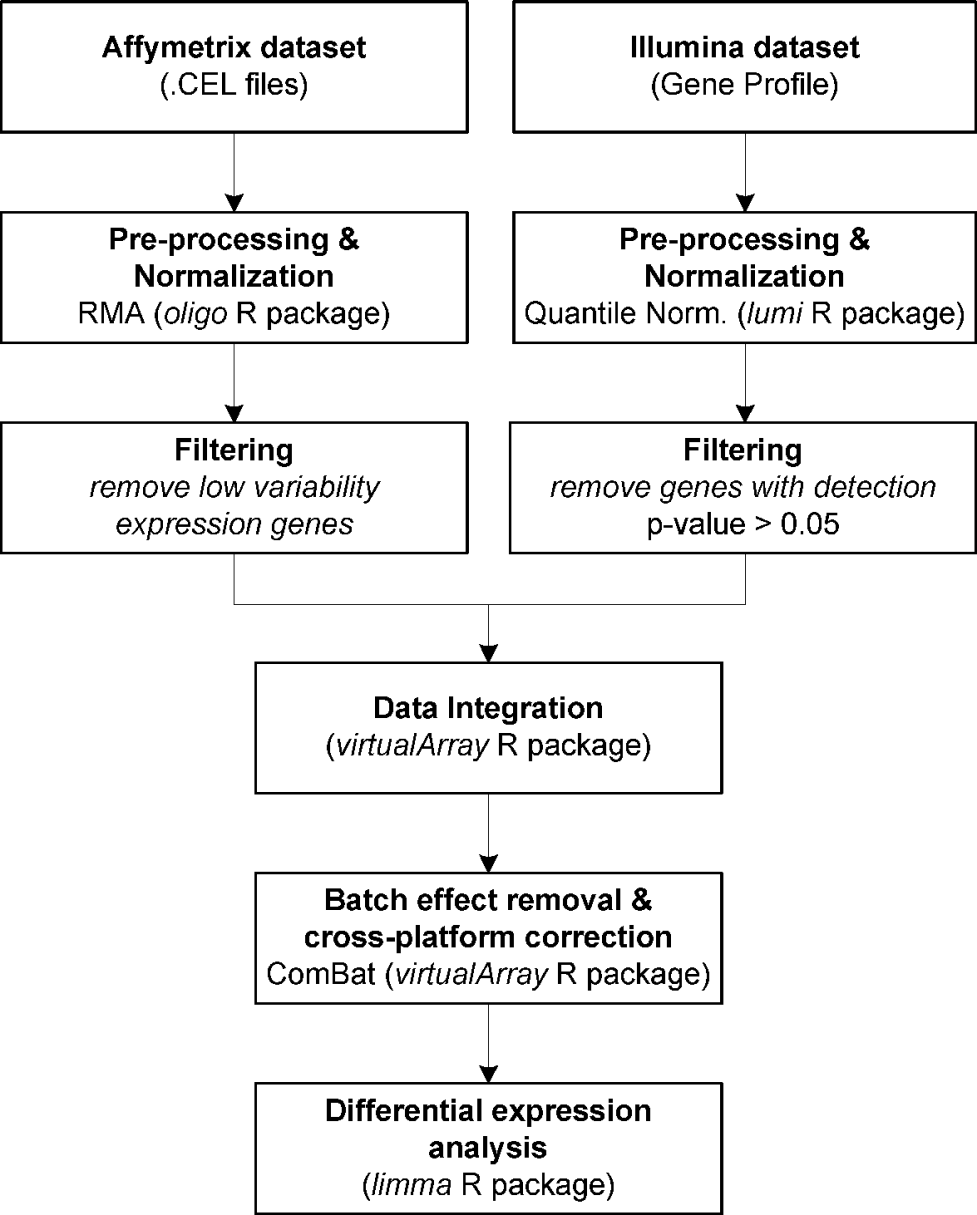
Workflow of the whole integrated meta-analysis for integration of Affymetrix/Illumina expression data from PDAC datasets.

All data processing and integration procedures were performed using the R statistical programming language. The data were integrated by adapting the scheme from Turbull et al. [13] with particular stages for the analysis of PDAC data in Affymetrix and Illumina. The workflow of the proposed meta-analysis is shown in Fig 1. More specifically, hybridization data from Affymetrix (Cohort 1) were first normalized using Robust Multi-array Average (RMA) analysis from the Bio-conductor R package *oligo* [23]. In the same way, Illumina expression data (Cohort 2) was pre-processed by applying Quantile Normalization (QN) from the R package *lumi* [24]. In both cases, genes with low variability expression values were discarded to reduce false-positive rates.

Data from both platforms were integrated with the *virtualArray* software R package [25]. This software allows data from different microarray platforms to be merged by considering several batch effect removal and cross-platform correction methods. Specifically, the data were integrated using the empirical Bayes method (ComBat) [19]. The ComBat method merges the information from several genes with similar expression distributions in each dataset to estimate the average and variance in each of those genes [26]. From the integrated data, those genes most likely to be differentially expressed in PDAC patients *versus* controls were selected by analyzing the gene expression microarray data with the linear models for microarray data (*limma*) software package [27]. The R script for the integrative meta-analysis is included as S1 File.

To validate the selected genes as PDAC biomarkers, a leave-one-out cross-validation (LOOCV) was performed with them. In this validation, one sample is consecutively discarded from the initial dataset, leaving a temporary training set and one left-out sample (test sample). This validation procedure is extensively used to assess a prediction model when no validation dataset is available.

Finally, we performed a GO enrichment analysis over the set of newly discovered genes after meta-analysis. For this purpose, an enrichment test using the Kolmogorov-Smirnov (KS) statistical test was carried out from *topGO* Bioconductor-R package. This analysis identified those biological functions and process that are shared by the differentially expressed genes.

## Results

### Patient characteristics

Our study included two independent cohorts of patients. The first group (1) included 18 PDAC patients and the independent cohort (2) comprised 36 PDAC patients. Table 1 shows the most relevant clinical characteristics of the patients from each cohort.

**Table 1:** Characteristics of both Cohort 1 and Cohort 2 groups of PDAC patients.

	Cohort 1 (n = 18)	Cohort 2 (n = 36)
Characteristic	N°. case (%)	N°. case (%)
*Sex*		
Male	9 (50%)	24 (67%)
Female	9 (50%)	12 (33%)
*Age Mean±SD*	61.4±10.7	60.0±7.7
Maximum	76	73
Minimum	37	42
*Pancreatitis*		
Yes	0 (0%)	2 (5.6%)
No	18 (100%)	34 (94.4%)
*Diabetes*		
Yes	7 (38.9%)	14 (38.9%)
No	11 (61.1%)	22 (61.1%)
*Stage*		
I	0 (0%)	0 (0%)
II	0 (0%)	0 (0%)
III	6 (33.3%)	0 (0%)
IV	12 (66.7%)	36 (100%)

Cohort 1, selected from our previous study [22], consisted of 9 men (50%) and 9 women (50%) with a mean age of 61.4 (range 37–76). None of the patients had a history of chronic pancreatitis, but 7 (38.9%) had a history of type II diabetes mellitus prior to being diagnosed with PDAC. At the time of diagnosis, 12 patients (66.6%) had stage IV tumors and 6 (33.4%) presented stage III tumors.

The PDAC patients from Cohort 2 comprised 24 men (67%) and 12 women (33%) with a mean age of 60.0 (range 42–73). Only 2 patients (5.6%) had a history of chronic pancreatitis, however, 14 patients (38.9%) had a history of type II diabetes mellitus. At the time of diagnosis, all patients had stage IV tumors.

Also, 18 healthy subjects were also included in the study. The control group consisted of 10 men (55.6%) and 8 women (44.4%) with a mean age of 60.4 (age range 35–74 years); none of these subjects had a history of either chronic pancreatitis or type II diabetes mellitus.

### Differential gene expression profiling of PBMCs from PDAC patients

After normalization and integration using *virtual Array*, the statistical differences in gene expression between the PDAC patients and healthy controls were analyzed with *limma* software. The data were integrated following the ComBat approach in order to reduce the batch effect produced amongst arrays. The effectiveness of the ComBat method in our integration for batch removal can be confirmed according to the comparative boxplots and density plot at S1 Fig. From this meta-analysis, 72 genes were consistently identified as being differentially expressed (*p*<0.01) with at least a 1.5-fold differential expression between the groups. Of these 72 genes, 39 were overexpressed and 33 repressed (Table 2 and S1–S3 Tables).

**Table 2:** Coincident genes in the three analyzes: Affymetrix, Illumina and integrated meta-analysis.

Gene	Gene description	ENTREZ^a^	FC^b^	adj.P.Val
*FAIM3*	Fas apoptotic inhibitory molecule 3	9214	- 2.17	4.59E-11
*IRAK3*	interleukin-1 receptor-associated kinase 3	11213	1.84	4.59E-11
*DENND2D*	DENN/MADD domain containing 2D	79961	- 1.67	1.08E-09
*PLBD1*	phospholipase B domain containing 1	79887	1.67	1.50E-09
*AGPAT9*	1-acylglycerol-3-phosphate O-acyltransferase 9	84803	1.58	1.47E-08

^a^Entrez Gene Name.

^b^Fold change.

**Fig 2:**
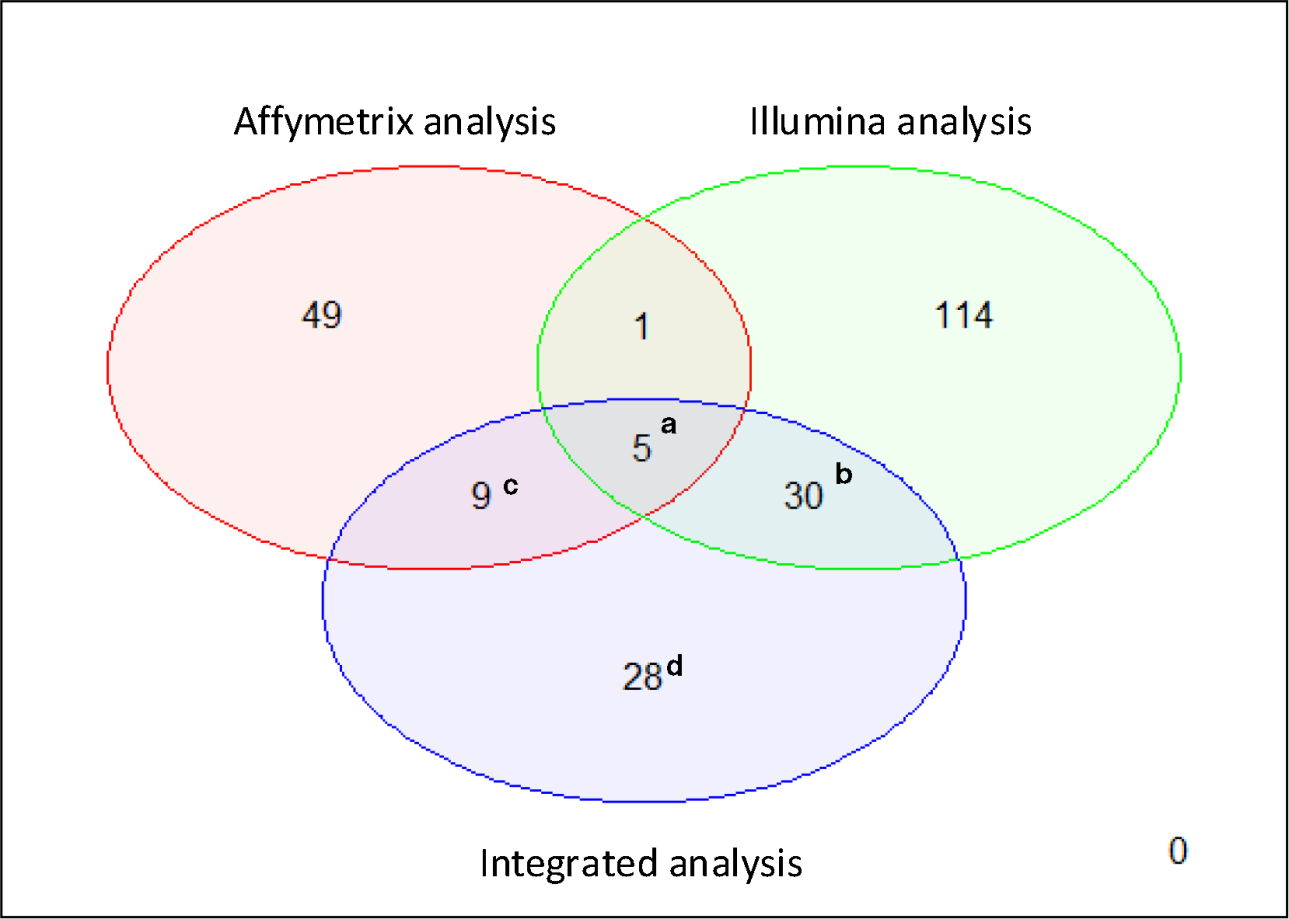
Comparison of individual analysis by technology with integrated analysis. **a** Coincident genes in the three analyzes: Affymetrix, Illumina and integrated meta-analysis (Table 2). **b** Remaining differentially expressed genes in individual Illumina and the integrative meta-analysis (S1 Table). **c** Remaining differentially expressed genes in individual Affymetrix and the integrative meta-analysis (S2 Table). **d** Differentially expressed genes in the integrative meta-analysis but not in individual analysis (gained genes) (S3 Table).

**Fig 3:**
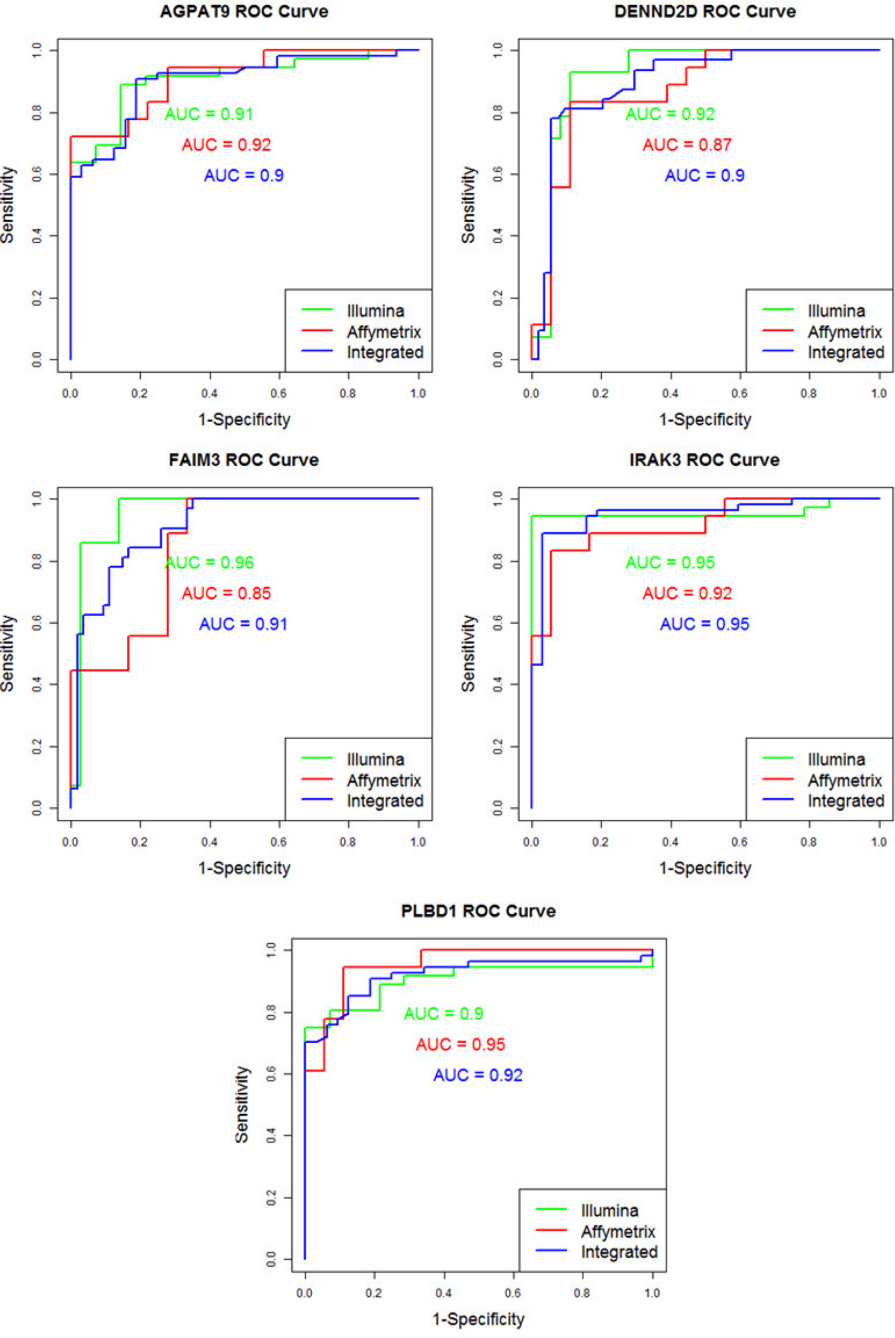
ROC Curves for the 5 genes commonly expressed: *FAMI3*, *IRAK3*, *DENND2D*, *PLBD1* and *AGPAT9*. Curves are provided for both Illumina and Affymetrix individual analyses as well as our integrative meta-analysis. The Area Under the Curve (AUC) metrics are also provided for each curve.

The meta-analysis findings were also compared with those obtained by individual analyses in both datasets to evaluate bias and reproducibility across the microarray studies. As a result, 14 genes already identified in the Affymetrix study were also highlighted by our meta-analysis, whereas 35 genes were shared with the Illumina study (Fig 2). Five of these genes were consistently identified by the three studies (Affymetrix, Illumina and integrated meta-analysis) (Table 2). Also ROC curves and areas under the curve (AUC) metrics were calculated for those 5 genes (Fig 3). Finally, a leave-one-out cross-validation was performed over these genes to demonstrate their predictive power. The accuracy values (sensitivity/specificity) obtained from this cross-validation is shown in Table 3.

**Table 3:** Sensitivity and specificity values for the selected genes after a leave-one-out cross-validation (LOOCV) process.

Gene	Sensitivity	Specificity
*FAIM3*	0.889	0.75
*IRAK3*	0.87	0.969
*DENND2D*	0.944	0.75
*PLBD1*	0.852	0.813
*AGPAT9*	0.889	0.813

Additionally, 28 gained genes were found. Gained genes are those identified as differentially expressed in the meta-analysis but not in the individual studies. These genes may be only weakly relevant individually but provide more consistent expression patterns when several datasets are integrated [28]. In order to determine the predictive power of these gained genes, their individual ROC curves were also studied (S3 Fig). Therefore, each individual gene was able to discriminate between PDAC patients and healthy controls with an average sensitivity and specificity of 74.8% and 73.3%, respectively. The same prediction analysis was performed combining the 5 commonly expressed genes as well as the 28 gained genes (S4 Fig). In this case, the sensitivity and specificity results reached the 100% and 94% for the 5 commons genes and 91% and 87% for the 28 gained genes.

Finally, we applied a GO enrichment analysis for the 28 gained genes. A total of 12 biological processes were found to be significant across these genes (Table 4).

**Table 4:** Shared Gene Ontology (GO) terms after the gene enrichment analysis applied over the 28 gained genes. The Kolmogorov-Smirnov statistical test was performed to determine their significance (*p*-value < 0.05).

GO ID	GO Term	Ontology	# Genes	*p*-value	Genes
GO:0044237	cellular metabolic process	BP	12	0.011	*ANXA3; HP; ITGB3;NLRC4; PLSCR1;RASGRP1; RPS28; S100A12; SH2D1B; ST6GAL1; TXK; VAMP2*
GO:0044763	single-organism cellular process	BP	23	0.015	*ANXA3; BPI; CD177; CLEC2D; CLEC4E; DYSF; GPR141; HP; ITGB3; LCN2; MS4A1; MYL9; NLRC4; PLSCR1; RASGRP1; RPS28; S100A12; SH2D1B; SLC38A1; SORT1; ST6GAL1; TXK; VAMP2*
GO:0050776	regulation of immune response	BP	5	0.023	*NLRC4; PLSCR1; RASGRP1; SH2D1B; TXK*
GO:0044710	single-organism metabolic process	BP	6	0.037	*HP; PLSCR1; RASGRP1; S100A12; ST6GAL1; VAMP2*
GO:0006139	nucleobase-containing compound metabolic process	BP	7	0.043	*ANXA3; NLRC4; PLSCR1; RASGRP1; RPS28; S100A12; TXK*
GO:0006725	cellular aromatic compound metabolic process	BP	7	0.043	*ANXA3; NLRC4; PLSCR1; RASGRP1; RPS28; S100A12; TXK*
GO:0006807	nitrogen compound metabolic process	BP	7	0.043	*ANXA3; NLRC4; PLSCR1; RASGRP1; RPS28; S100A12; TXK*
GO:0034641	cellular nitrogen compound metabolic process	BP	7	0.043	*ANXA3; NLRC4; PLSCR1; RASGRP1; RPS28; S100A12; TXK*
GO:0034645	cellular macromolecule biosynthetic process	BP	7	0.043	*ANXA3; NLRC4; PLSCR1; RPS28; S100A12; ST6GAL1; TXK*
GO:0044249	cellular biosynthetic process	BP	7	0.043	*ANXA3; NLRC4; PLSCR1; RPS28; S100A12; ST6GAL1; TXK*
GO:0046483	heterocycle metabolic process	BP	7	0.043	*ANXA3; NLRC4; PLSCR1; RASGRP1; RPS28; S100A12; TXK*
GO:1901360	organic cyclic compound metabolic process	BP	7	0.043	*ANXA3; NLRC4; PLSCR1; RASGRP1; RPS28; S100A12; TXK*

## Discussion

Affymetrix GeneChips and Illumina BeadChips are the main platforms used for gene expression microarrays. However, non-trivial systematic bias (batch effects) can occur in both making it necessary to use appropriate correction methods when integrating the datasets from the two technologies [13]. Also, differences in sequences and the number of probes make it even more difficult to integrate their datasets. Consequently, a complex integration method is mandatory in order to successfully perform consistent meta-analyses.

Several batch correction and cross-platform normalization approaches have been proposed for this purpose, including mean-centering (MC), DWD, and empirical Bayesian (ComBat) method. Even though the three proposals were compared for this integrated meta-analysis, the ComBat approach was finally selected. ComBat has been highly recommended in the literature due to its reduced computational cost and the fact it is independent of sample size [26]. It has also proven to be useful in reducing inter-platform variance, outperforming other similar approaches such as DWD or MC (see S2 Fig) [13]. Nevertheless, it is important to highlight that this methodology is still being carefully revised. Thus, novel alternatives are continually being proposed in the literature trying to correct bias more efficiently among disease samples, for instance, using co-normalization of control samples [29] or combining with other normalization approach like LOESS, SVN or QN [30,31].

Also, given that the multi-platform integration with virtualArray is based on ExpressionSet format, the proposed meta-analysis could be easily extended to integratditionale other data sources like RNA-Seq from next-generation sequencing technologies [25]. In fact, the widely used RNA-Seq expression analysis with the R package DESeq [32] already applies variance-stabilizing transformation to convert and normalize raw count values to ExpressionSet format.

Other similar meta-analyses have already been carried out to identify biomarkers in pancreatic cancers from several microarray datasets [18]. Nevertheless, these solutions provide DEGs merely by statistically determining the intersection between datasets. In contrast, a more thorough integrative approach including batch correction and cross-platform normalization is proposed in this work.

After this integration, 5 genes, namely Fas apoptotic inhibitory molecule 3 (*FAIM3* or *TOSO*), IL-1 Receptor-Associated Kinase 3 (*IRAK3*), DENN/MADD Domain Containing 2D gene (*DENND2D*), Phospholipase B Domain Containing 1 (*PLBD1*) and 1-Acylglycerol-3-Phosphate O-Acyltransferase 9 (*AGPAT9* or *MAG-1*), were identified as being commonly differentially expressed by the individual analyses in Affymetrix and Illumina as well as by the integrated meta-analysis. These genes were shown to be potential predictors for PDAC diagnosis given they showed areas under the curve (AUC) metrics higher than 0.9 for their corresponding ROC curves (Fig 3). Therefore, these genes were considered reliable targets since they showed consistent differential expression in the integrated analysis and higher predictive metrics. In fact, *IRAK-3* has already been studied and validated by RT-qPCR in our previous study using Affymetrix [22]. Also, the other three genes validated in Affymetrix, namely *ANKRD22*, *CLEC4D* and *VNN1* were similarly identified in the proposed meta-analysis.

More specifically, our results showed downregulation of the gene *FAIM3*, which plays an important role in the immune system as it encodes an Fc receptor for immunoglobulins (Ig), M. Fc receptors specifically bind to the Fc region of Igs to mediate the unique functions of each class [33,34]. The expression of FAIM3 is reported in peripheral blood leukocytes and detected in high levels in chronic lymphocytic leukemia cells [35]. It has been demonstrated that a decrease in *FAIM3* expression results in increased apoptosis, however, increased *FAIM3* expression resulting from CD25 antibody treatment protects T cells from IL-2-mediated activation-induced cell death (AICD) [36] underlining an involvement in the immune process.

The upregulation of the gene *IRAK3* may provide a clue about the mechanisms leading to immune evasion by tumor cells. This gene is expressed in monocytes and macrophages [37] and can be triggered by Toll-like receptors (TLRs) [38,39], which are expressed in various types of cancer [40]. Overall, *IRAK3* activation leads to immunosuppression [41] and allows the communication between tumor cells and macrophages facilitating cancer progression and a favorable microenvironment for the tumor [42,43]. In fact, monocytes from chronic myeloid leukemia and metastatic cancer patients present *IRAK3* upregulation, leading to tumor formation and growth [44]. In this sense, a study with mouse models carried out by Rothschild et al. [45] demonstrated the connection between *IRAK3* expression and both inflammation and colorectal cancer.

The *DENND2D* gene, another modified gene related to the immune system, has been suggested as a tumor suppressor gene [46]. DENN-domain proteins are differentially expressed in normal and neoplastic cells and regulate Rab GTPases, which play important roles in differentiation, proliferation processes, and regulation of cancer cells, among other things [47,48]. *DENND2D* has been proposed to suppress the tumorigenicity and proliferation of lung cancer cells [49,50]. In addition, the DENND2D mRNA expression level has been found to be significantly lower in esophageal squamous cell carcinoma tissues, hepatocellular carcinoma [51], lung cancers, immortalized bronchial epithelial cell lines and other precancerous lesions [46,50].

In our study, the *PLBD1* gene expression level coding was shown to be elevated in PDAC patients. This gene is highly expressed in neutrophils and monocytes [52] and members of this family have been related to antibacterial defense [53].

Metastatic ability is one of the major problems associated with pancreatic cancer. In this regard, our study reveals the overexpression of the *AGPAT9* gene, which has been associated with the metastatic process in lung cancer [54,55]. Various important functions of *AGPAT9* have been described in this metastatic process. First, *AGPAT9* is involved in the adaptation to the microenvironment, regulating the metabolism and hypoxia, and contributing to vascular development increasing the expression of VEGF. Furthermore, *AGPAT9* is involved in mTOR pathway activation which is key in the metastatic process [56].

Additionally, 28 novel gained genes were found to have more robust patterns in the meta-analysis than in individual studies, making them statistically more significant as possible biomarkers (S3 Table).

Upregulated Annexin A3 (*ANXA3*) and downregulated Membrane-Spanning 4-Domains Subfamily A Member 1 (*MS4A1*) were novel gained genes discovered using this technique. These results are supported by Baine et al., who also included both genes as part of a predictor set of biomarkers in the PBMC of PC patients [57]. Also, Haptoglobin (*HP*) and Lipocalin 2 (*LCN2*) appeared upregulated in this new set of genes. The presence of fucosylated *HP* in serum has been associated to many cancers including hepatocellular, gastric and colon cancers, but the highest incidence has been observed in PC, mainly at an advanced stage [58]. Increased *LCN2* levels have been related to the epithelial to mesenchymal transition [59] and proposed as a serum marker for familial PC [60]. Moreover, we observed the upregulation of other genes like CD177 Molecule (*CD177*), Phospholipid Scramblase 1 (*PLSCR1*), Secretory Leukocyte Peptidase Inhibitor (*SLPI*), S100 Calcium Binding Protein A12 (*S100A12*) and Integrin Beta 3 (*ITGB3*), all of them related to the development of different gastrointestinal tumors [61–66].

It is also noteworthy that all the novel genes that appeared downregulated are associated with the immune response: Granulysin (*GNLY*) functions as a chemotactic for T-lymphocytes, monocytes and other inflammatory cells [67]; Natural Killer Cell Granule Protein 7 (*NKG7*) is expressed in several cell types, including NK and T-cells [68]; C-type Lectin Domain Family 2, Member D (*CLEC2D*) is a receptor present in NK cells [69]; TXK Tyrosine Kinase (*TKX*) takes part in the Th1 cytokine production and is implicated in the adaptive immune response [70]; and RAS Guanyl Releasing Protein 1 (*RASGRP1*) has been found to play an important role in T-cell development [71].

## Conclusions

An innovative meta-analysis has been performed to combine two gene expression datasets containing PDAC data and identify robust DEGs in these patients. Integrative meta-analyses have been shown to be powerful tools for identifying more robust DEGs when working with different data sources. Thus, an empirical Bayes approach (ComBat) has been employed in this study to integrate data from two different microarray technologies, namely Affymetrix GeneChip® Human Gene ST 1.0 Arrays and Illumina HumanHT-12 v4 Expression BeadChip, removing the batch effect between technologies and increasing the statistical significance of the subsequent analysis. The integrative analysis has confirmed the DEGs previously published for the Affymetrix data but has also located a set of gained genes that were not robust enough to be identified in the individual analyses. Thus, most of the genes identified have already been annotated as biomarkers in PDAC whereas other gained genes observed in this meta-analysis have also been related to several gastroenterological cancers. The proposed method has therefore been proven useful for more in-depth analysis of heterogeneous expression datasets, improving the identification of DEGs and discovering novel potential biomarkers for diagnosing PDAC. Future RT-qPCR studies will be performed to validate the gained genes that are considered interesting for this purpose. The proposed meta-analysis is also planned to be extended using RNA-Seq data from additional PDAC samples.

## Supporting information

S1 File R Script including code used to obtain results showed in this paper for the integrative meta-analysis. (ZIP)

S1 Fig Graphical analysis of the batch analysis removal. (A) Boxplots for the gene expression distributions in Cohort 1 (Affymetrix), Cohort 2 (Illumina) and healthy controls before applying ComBat batch removal. (B) Same boxplots after ComBat batch removal. The distributions show the normalization and reduction of technical differences between cohorts. (C) Density plot and standard deviation of expression across arrays after integration. The red dotted line indicates the median of the standard deviation. An approximately horizontal red line indicates an effective removal of bias and batch effects among arrays.
(PNG)

S2 Fig Comparison of batch removal method. (A) Boxplots and standard deviation of expression after applying the mean-centering (MC) method. (B) Boxplots and standard deviation after applying the distance discretization method. Although differences cannot be appreciated in boxplots, the median of the standard deviation (red dotted line) indicated a slightly better linearity in ComBat method (see S1 Fig). Additionally, the median standard deviation is also clearly lower for ComBat batch removal.
(PNG)

S3 Fig Individual ROC curve for the 28 gained genes. ROC curves for the gained genes. The area under the curve (AUC) is performed to estimate the predictive power of each gene. A cut-off is determined to optimize the discrimination between PDAC patients and healthy controls. The corresponding specificity and sensitivity values are calculated accordingly.
(PDF)

S4 Fig ROC curves for combined genes. (A) The ROC curve and its corresponding AUC, sensitivity and specificity are obtained for the combination of the 5 genes shared by the three studies (Illumina, Affymetrix and meta-analysis). (B) The ROC curve as well as AUC, sensitivity and specificity values is also obtained for the combination of the 28 gained genes.
(PNG)

S1 Table Remaining differentially expressed genes in individual Illumina and the integrative meta-analysis. (PDF)

S2 Table Remaining differentially expressed genes in individual Affymetrix and the integrative meta-analysis. (PDF)

S3 Table Differentially expressed genes in the integrative meta-analysis but not in individual analysis (*gained* genes). (PDF)
