# Supplementary figures and images for: Integrative multi-platform meta-analysis of gene expression profiles in pancreatic ductal adenocarcinoma patients for identifying novel diagnostic biomarkers
Source: PLoS One. 2018 Apr 4;13(4):e0194844. doi: 10.1371/journal.pone.0194844 (PMC5884535; doi:10.1371/journal.pone.0194844)

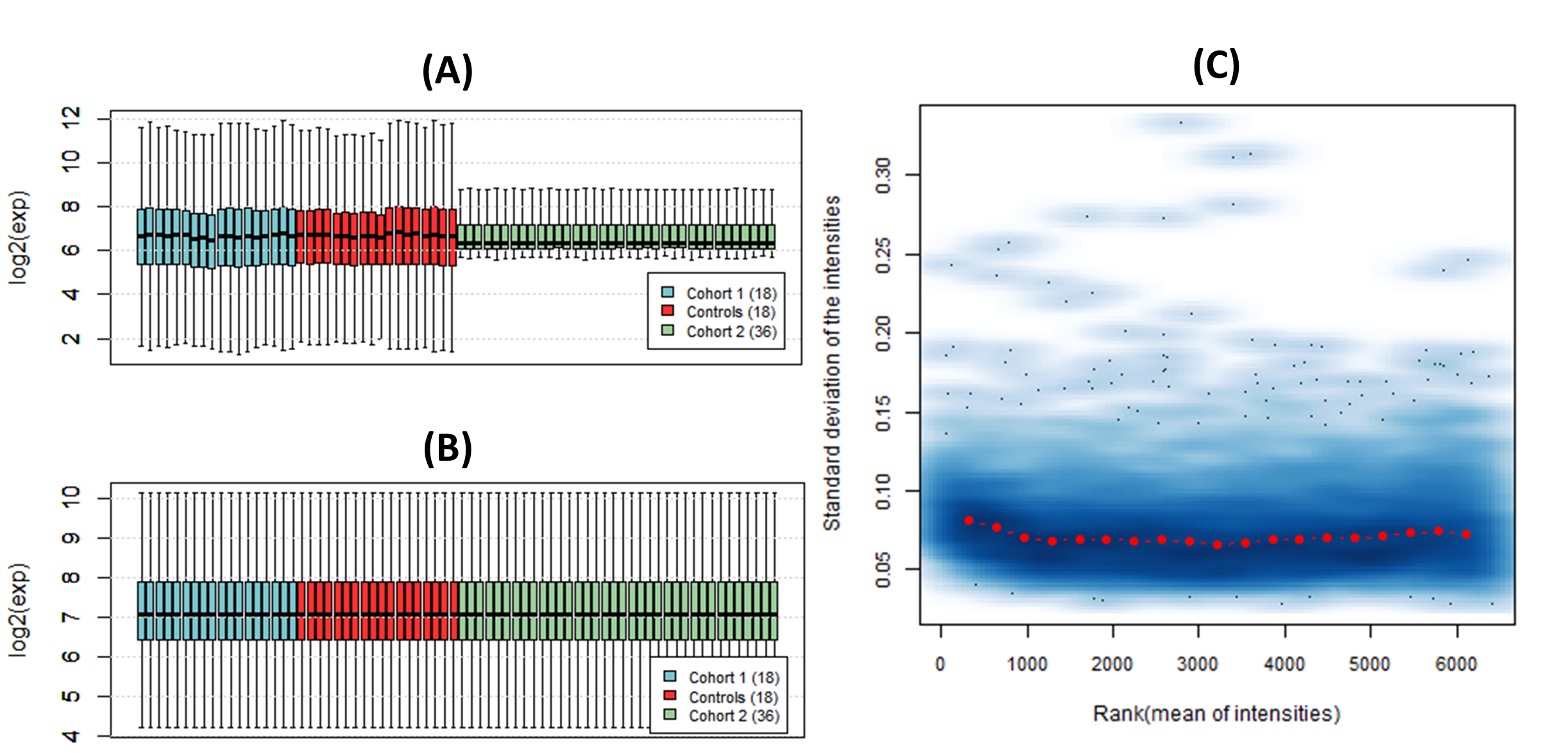

Supplement: S1 Fig — (A) Boxplots for the gene expression distributions in Cohort 1 (Affymetrix), Cohort 2 (Illumina) and healthy controls before applying ComBat batch removal. (B) Same boxplots after ComBat batch removal. The distributions show the normalization and reduction of technical differences between cohorts. (C) Density plot and standard deviation of expression across arrays after integration. The red dotted line indicates the median of the standard deviation. An approximately horizontal red line indicates an effective removal of bias and batch effects among arrays. (PNG) [file pone.0194844.s002.png]

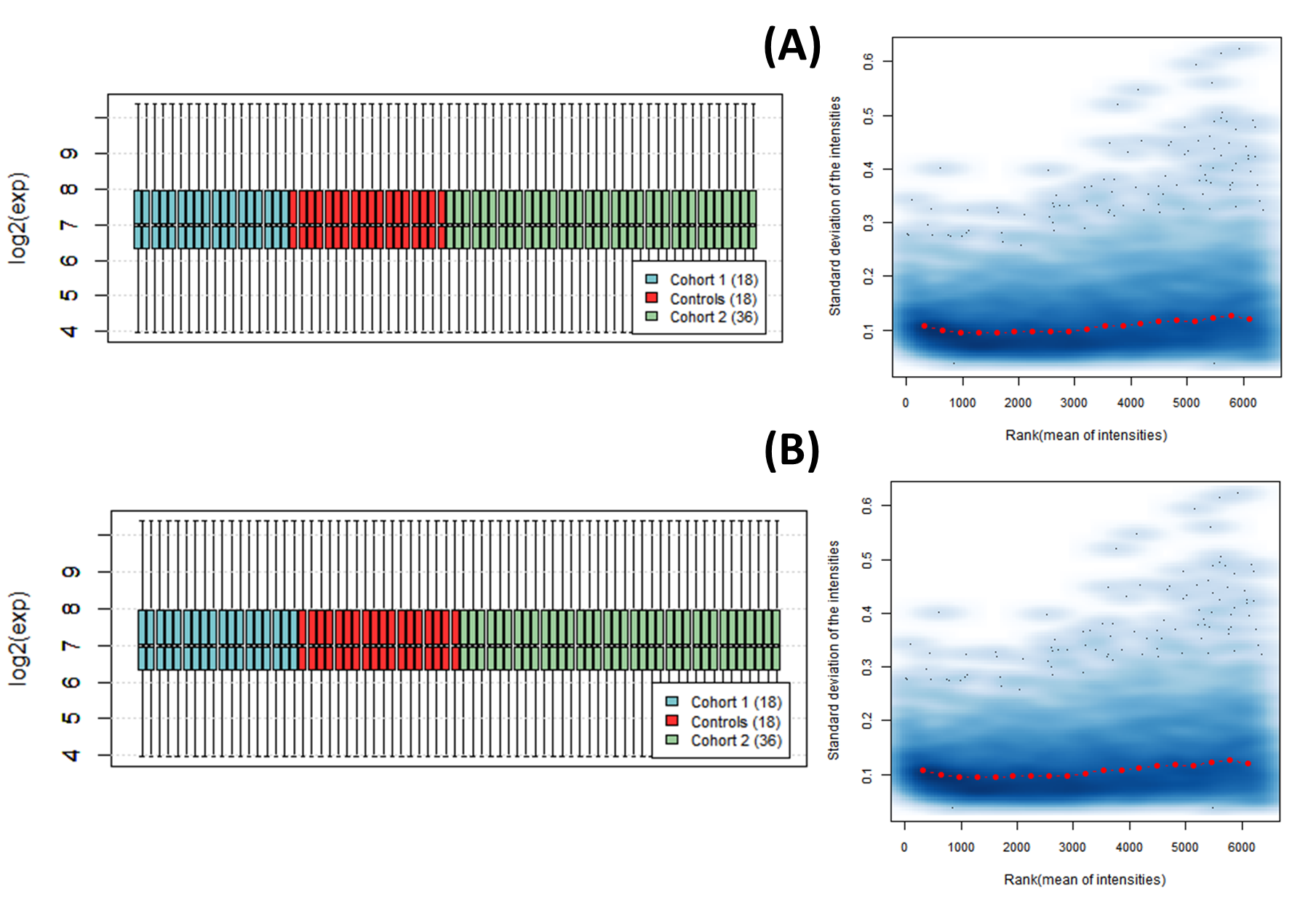

Supplement: S2 Fig — (A) Boxplots and standard deviation of expression after applying the mean-centering (MC) method. (B) Boxplots and standard deviation after applying the distance discretization method. Although differences cannot be appreciated in boxplots, the median of the standard deviation (red dotted line) indicated a slightly better linearity in ComBat method (see S1 Fig). Additionally, the median standard deviation is also clearly lower for ComBat batch removal. (PNG) [file pone.0194844.s003.png]

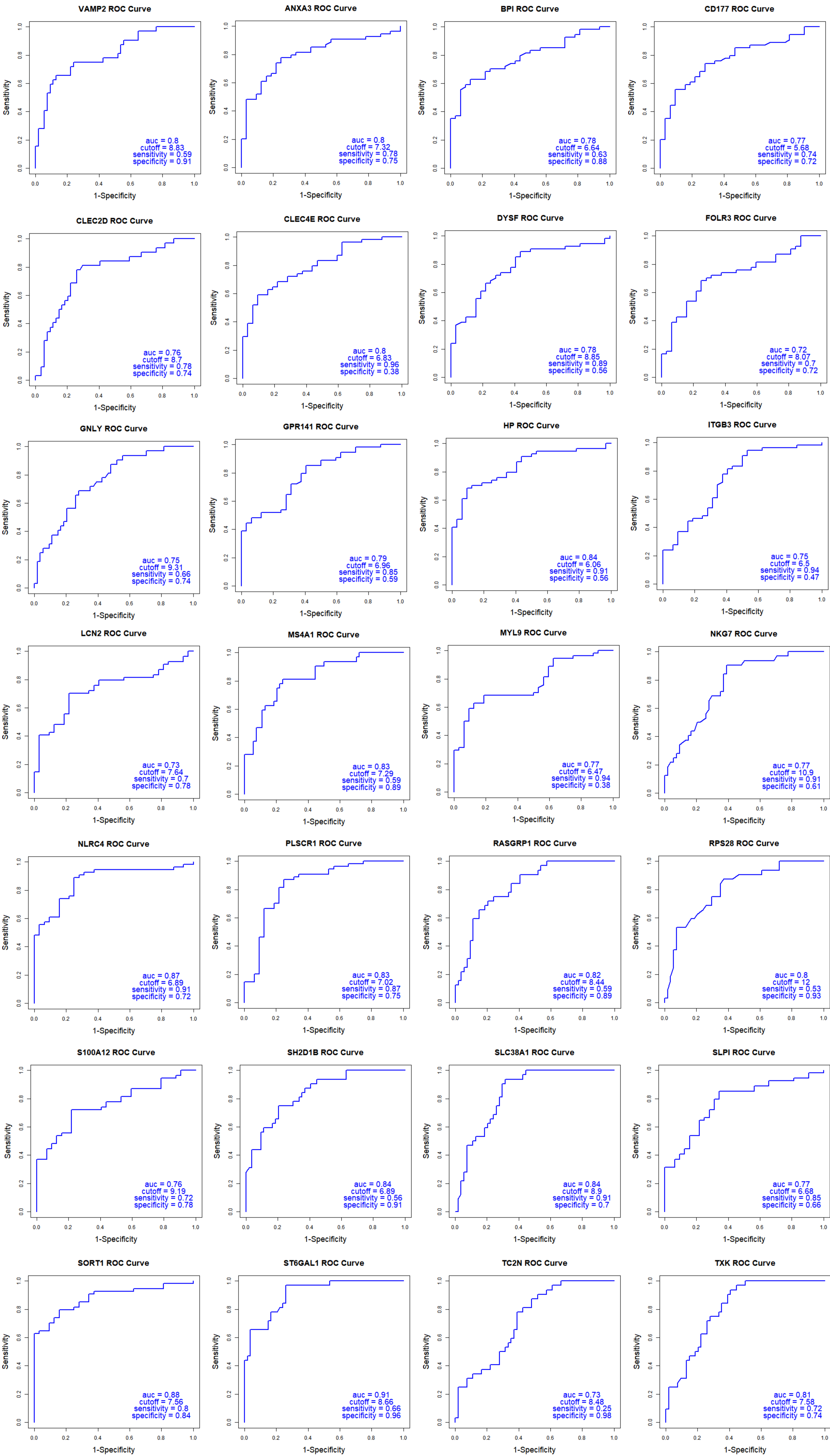

Supplement: S3 Fig — ROC curves for the gained genes. The area under the curve (AUC) is performed to estimate the predictive power of each gene. A cut-off is determined to optimize the discrimination between PDAC patients and healthy controls. The corresponding specificity and sensitivity values are calculated accordingly. (PDF) [file pone.0194844.s004.pdf]

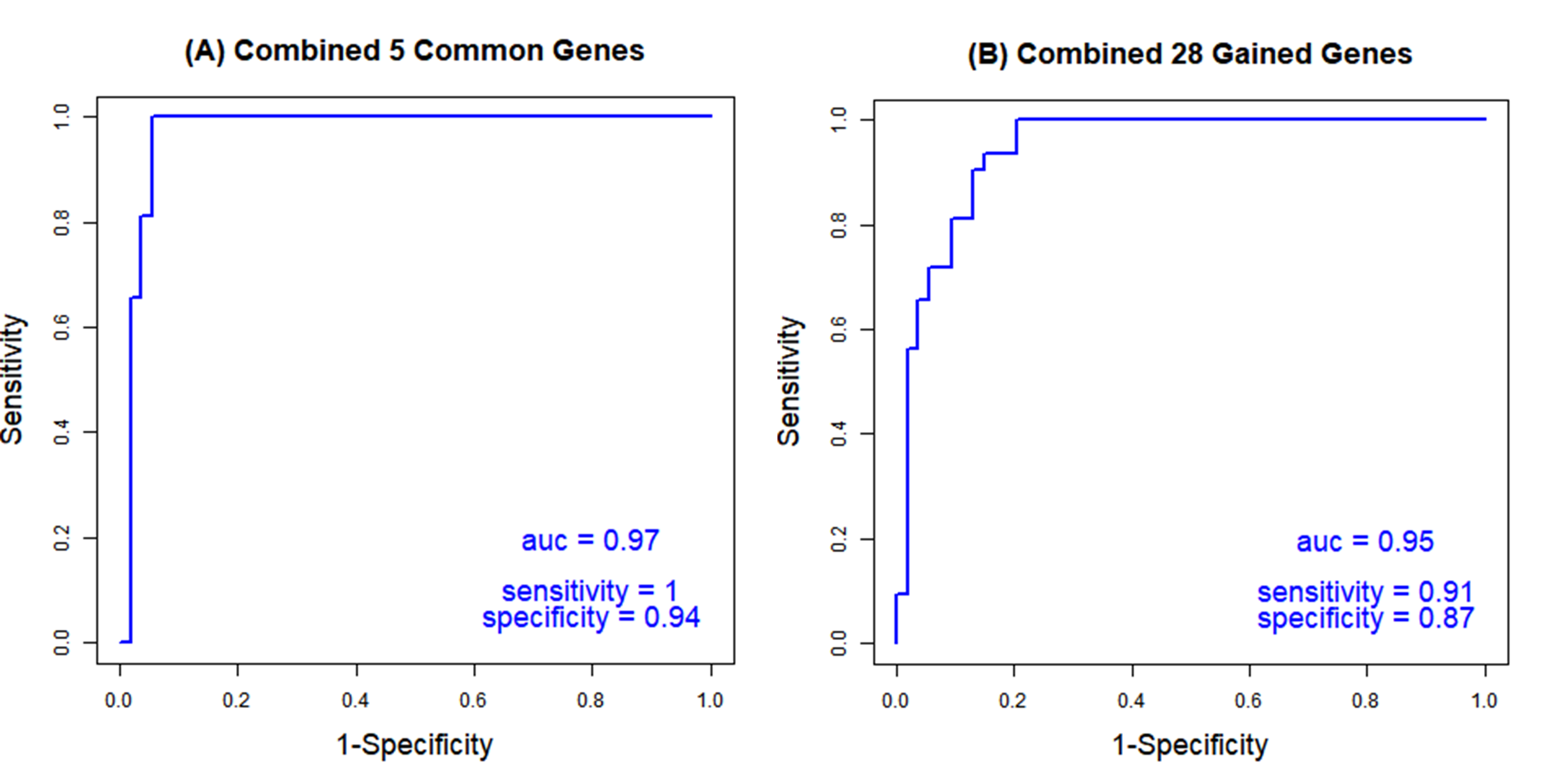

Supplement: S4 Fig — (A) The ROC curve and its corresponding AUC, sensitivity and specificity are obtained for the combination of the 5 genes shared by the three studies (Illumina, Affymetrix and meta-analysis). (B) The ROC curve as well as AUC, sensitivity and specificity values is also obtained for the combination of the 28 gained genes. (PNG) [file pone.0194844.s005.png]
