# Supplementary material for: Integrative multi-platform meta-analysis of gene expression profiles in pancreatic ductal adenocarcinoma patients for identifying novel diagnostic biomarkers
Source: PLoS One. 2018 Apr 4;13(4):e0194844. doi: 10.1371/journal.pone.0194844 (PMC5884535; doi:10.1371/journal.pone.0194844)
Supplement: S1 Table — (PDF) [file pone.0194844.s006.pdf]

S1 Table. Remaining differentially expressed genes in individual Illumina and the integrative meta-analysis.						
**ENTREZ**	**SYMBOL**	**GENENAME**	**logFC**	**adj.P.Val**	**B**	**FC**
4318	MMP9	matrix metallopeptidase 9	1.12	1.91E-04	3.49	2.18
3560	IL2RB	interleukin 2 receptor, beta	-0.98	5.50E-07	10.88	-1.96
3575	IL7R	interleukin 7 receptor	-0.96	5.80E-07	10.71	-1.96
3945	LDHB	lactate dehydrogenase B	-0.96	5.64E-05	5.01	-1.92
1236	CCR7	chemokine (C-C motif) receptor 7	-0.95	3.79E-07	11.39	-1.92
353514	LILRA5	leukocyte immunoglobulin-like receptor, subfamily A, member 5	0.91	5.69E-06	7.83	1.89
23569	PADI4	peptidyl arginine deiminase, type IV	0.88	8.34E-07	10.23	1.83
973	CD79A	CD79a molecule, immunoglobulin-associated alpha	-0.86	1.10E-05	7.08	-1.82
51176	LEF1	lymphoid enhancer-binding factor 1	-0.86	5.50E-07	10.82	-1.82
6191	RPS4X	ribosomal protein S4, X-linked	-0.83	6.19E-04	1.98	-1.79
3820	KLRB1	killer cell lectin-like receptor subfamily B, member 1	-0.8	2.71E-07	11.89	-1.75
5583	PRKCH	protein kinase C, eta	-0.76	5.18E-08	13.9	-1.69
2205	FCER1A	Fc fragment of IgE, high affinity I, receptor for; alpha polypeptide	-0.74	8.77E-07	10.1	-1.67
3674	ITGA2B	integrin, alpha 2b	0.74	8.40E-05	4.56	1.67
916	CD3E	CD3e molecule, epsilon	-0.74	8.77E-07	10.1	-1.67
7850	IL1R2	interleukin 1 receptor, type II	0.73	5.43E-04	2.15	1.66
939	CD27	CD27 molecule	-0.72	5.18E-08	13.95	-1.64
3702	ITK	IL2-inducible T-cell kinase	-0.7	1.41E-06	9.56	-1.61
2113	ETS1	v-ets avian erythroblastosis virus E26 oncogene homolog 1	-0.69	1.11E-03	1.22	-1.61
762	CA4	carbonic anhydrase IV	0.67	2.92E-06	8.64	1.60
10410	IFITM3	interferon induced transmembrane protein 3	0.67	2.80E-03	0.15	1.59
1462	VCAN	versican	0.66	1.09E-04	4.23	1.58
340205	TREML1	triggering receptor expressed on myeloid cells-like 1	0.66	7.55E-04	1.7	1.58
6194	RPS6	ribosomal protein S6	-0.66	1.59E-04	3.74	-1.59
3101	HK3	hexokinase 3	0.65	4.33E-06	8.14	1.57
2162	F13A1	coagulation factor XIII, A1 polypeptide	0.64	3.38E-03	-0.09	1.55
6556	SLC11A1	solute carrier family 11, member 1	0.62	9.55E-04	1.41	1.54
5610	EIF2AK2	eukaryotic translation initiation factor 2-alpha kinase 2	0.62	5.07E-05	5.19	1.54
919	CD247	CD247 molecule	-0.6	5.64E-07	10.76	-1.52
3003	GZMK	granzyme K	-0.6	1.78E-04	3.59	-1.52
