# Supplementary material for: Integrative multi-platform meta-analysis of gene expression profiles in pancreatic ductal adenocarcinoma patients for identifying novel diagnostic biomarkers
Source: PLoS One. 2018 Apr 4;13(4):e0194844. doi: 10.1371/journal.pone.0194844 (PMC5884535; doi:10.1371/journal.pone.0194844)
Supplement: S2 Table — (PDF) [file pone.0194844.s007.pdf]

S2 Table. Remaining differentially expressed genes in individual Affymetrix and the integrative meta-analysis.						
**ENTREZ**	**SYMBOL**	**GENENAME**	**logFC**	**adj.P.Val**	**B**	**FC**
6279	S100A8	S100 calcium binding protein A8	0.93	8.20E-05	4.59	1.90
118932	ANKRD22	ankyrin repeat domain 22	0.86	6.22E-08	13.67	1.81
8876	VNN1	vanin 1	0.84	4.07E-07	11.29	1.79
2153	F5	coagulation factor V	0.77	5.22E-09	16.89	1.71
383	ARG1	arginase 1	0.71	3.45E-04	2.72	1.64
54674	LRRN3	leucine rich repeat neuronal 3	-0.71	7.24E-06	7.56	-1.64
64757	MARC1	mitochondrial amidoxime reducing component 1	0.66	5.50E-07	10.84	1.58
338339	CLEC4D	C-type lectin domain family 4, member D	0.6	1.55E-04	3.79	1.52
115352	FCRL3	Fc receptor-like 3	-0.59	5.84E-11	22.01	-1.49
