# Supplementary material for: Integrative multi-platform meta-analysis of gene expression profiles in pancreatic ductal adenocarcinoma patients for identifying novel diagnostic biomarkers
Source: PLoS One. 2018 Apr 4;13(4):e0194844. doi: 10.1371/journal.pone.0194844 (PMC5884535; doi:10.1371/journal.pone.0194844)
Supplement: S3 Table — (PDF) [file pone.0194844.s008.pdf]

S3 Table. Differentially expressed genes in the integrative meta-analysis but not in individual analysis (*gained* genes)

ENTREZ	SYMBOL	GENENAME	logFC	adj.P.Val	B	FC
57126	CD177	CD177 molecule	1.02	7.32E-04	1.75	2.02
6283	S100A12	S100 calcium binding protein A12	0.93	1.98E-04	3.44	1.91
3690	ITGB3	integrin, beta 3	0.84	6.56E-04	1.88	1.79
3240	HP	haptoglobin	0.82	2.37E-05	6.13	1.76
306	ANXA3	annexin A3	0.81	1.06E-04	4.25	1.76
931	MS4A1	membrane-spanning 4-domains, subfamily A, member 1	-0.8	1.64E-06	9.35	-1.72
671	BPI	bactericidal/permeability-increasing protein	0.73	7.89E-04	1.65	1.66
10578	GNLY	granulysin	-0.72	3.88E-04	2.57	-1.64
123036	TC2N	tandem C2 domains, nuclear	-0.72	1.85E-03	0.62	-1.64
6234	RPS28	ribosomal protein S28	-0.68	4.68E-05	5.28	-1.61
4818	NKG7	natural killer cell granule protein 7	-0.68	1.76E-04	3.61	-1.61
3934	LCN2	lipocalin 2	0.68	6.22E-03	-0.85	1.60
29121	CLEC2D	C-type lectin domain family 2, member D	-0.66	2.69E-04	3.02	-1.59
2352	FOLR3	folate receptor 3 (gamma)	0.66	1.94E-03	0.57	1.58
7294	TXK	TXK tyrosine kinase	-0.66	3.82E-05	5.53	-1.59
10125	RASGRP1	RAS guanyl releasing protein 1	-0.65	3.06E-06	8.56	-1.56
5359	PLSCR1	phospholipid scramblase 1	0.64	3.12E-07	11.68	1.56
6590	SLPI	secretory leukocyte peptidase inhibitor	0.64	8.20E-04	1.6	1.55
6480	ST6GAL1	ST6 beta-galactosamide alpha-2,6-sialyltranferase 1	-0.63	3.60E-10	19.99	-1.56
10398	MYL9	myosin, light chain 9, regulatory	0.62	2.33E-04	3.22	1.53
81539	SLC38A1	solute carrier family 38, member 1	-0.62	1.11E-05	7.06	-1.54
26253	CLEC4E	C-type lectin domain family 4, member E	0.61	2.92E-05	5.89	1.53
117157	SH2D1B	SH2 domain containing 1B	-0.61	5.50E-07	10.86	-1.52
6844	VAMP2	vesicle-associated membrane protein 2	-0.61	3.02E-06	8.6	-1.52
353345	GPR141	G protein-coupled receptor 141	0.6	4.77E-05	5.25	1.51
58484	NLRC4	NLR family, CARD domain containing 4	0.59	2.65E-07	11.95	1.51
8291	DYSF	dysferlin	0.59	6.61E-04	1.87	1.51
6272	SORT1	sortilin 1	0.59	9.80E-08	13.03	1.50
