# Supplementary material for: Integrative multi-platform meta-analysis of gene expression profiles in pancreatic ductal adenocarcinoma patients for identifying novel diagnostic biomarkers
Source: PLoS One. 2018 Apr 4;13(4):e0194844. doi: 10.1371/journal.pone.0194844 (PMC5884535; doi:10.1371/journal.pone.0194844)
Supplement: S1 File — (ZIP) [file pone.0194844.s001.zip › S1_File/virtualArray.tar!/virtualArray/inst/doc/virtualArray-016.pdf]

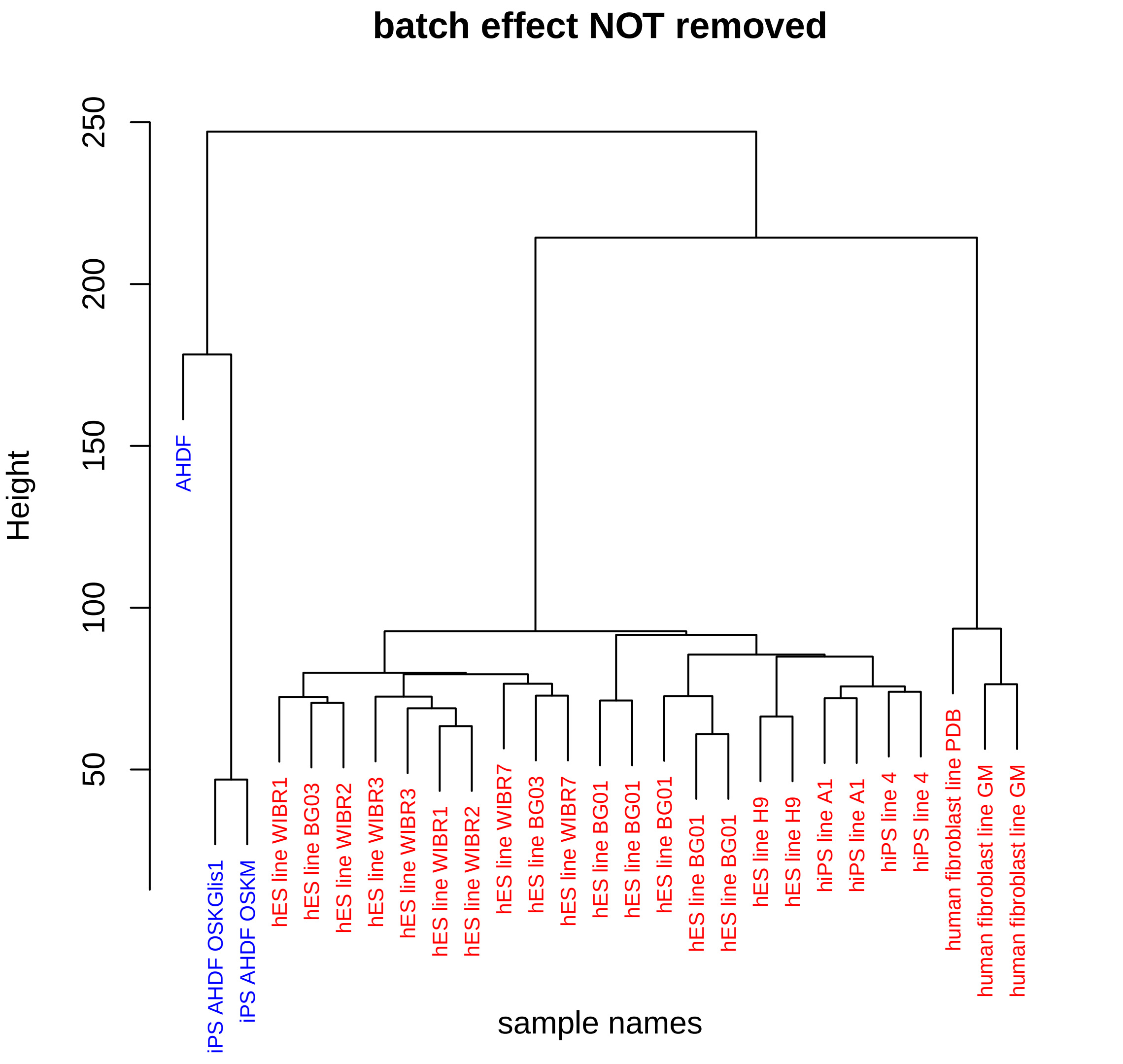
batch effect NOT removed
Height
250
200
150
100
50
sample names
AHDF
iPS AHDF OSKGlis1
iPS AHDF OSKM
hES line WIBR1
hES line BG03
hES line WIBR2
hES line WIBR3
hES line WIBR3
hES line WIBR1
hES line WIBR2
hES line WIBR7
hES line BG03
hES line WIBR7
hES line BG01
hES line BG01
hES line BG01
hES line BG01
hES line BG01
hES line H9
hES line H9
hiPS line A1
hiPS line A1
hiPS line 4
hiPS line 4
human fibroblast line PDB
human fibroblast line GM
human fibroblast line GM
