# Supplementary material for: Integrative multi-platform meta-analysis of gene expression profiles in pancreatic ductal adenocarcinoma patients for identifying novel diagnostic biomarkers
Source: PLoS One. 2018 Apr 4;13(4):e0194844. doi: 10.1371/journal.pone.0194844 (PMC5884535; doi:10.1371/journal.pone.0194844)
Supplement: S1 File — (ZIP) [file pone.0194844.s001.zip › S1_File/virtualArray.tar!/virtualArray/inst/doc/virtualArray.pdf]

virtualArray Package Vignette

Andreas Heider

11 August 2011

1 Introduction

Current tools for the analysis of microarray data only allow the comparison of datasets generated on the same platform and chip generation, and restrict meta-analysis studies to the evaluation of study results from different groups, rather than the direct comparison of the available raw datasets. The aim of the virtualArray package is to enable the combination of raw expression data of different microarray platforms into one "virtual array". Thus, the user may compare his own data to other datasets including public sources, regardless of the platform and chip generation used, or perform meta-analysis directly based on available raw datasets. The package generates a combined virtual array as a "ExpressionSet" object from different datasets by matching raw data entries based on probe, transcript, gene or protein identifiers. Redundancies, gaps, and batch effects are removed before proceeding with data analysis.

virtualArray consists of several subsequent functions, requiring minimal user input. Briefly, (1) raw data are loaded into R, (2) probe sets for each platform are annotated, (3) genes, proteins or transcripts common to all platforms are matched, including checks for redundancy or missing values, (4) data are compiled into a new "virtual array", (5) normalized and (6) subjected to batch effect removal using empirical Bayes methods [1] or another method of choice. The generated "virtual array" can than be directly analyzed in R/Bioconducor or exported for use in other suitable software (e.g. MeV).

There are essentially four modes of operation:

Firstly, the "virtualArrayCompile" function can integrate the major (but not all) human microarray platfroms in a default mode requiering minimal user input.

Secondly the "virtualArrayExpressionSets" function. This approach allows to integrate any kind of raw expression data that can be loaded into an ExpressionSet object in R/BioC. The downside in this case is that the user will have to deal with details such as log2-transformations, 16 bit - 20 bit transformations, assignment of correct annotations, etc.

Additionally, each of these two approaches can be used in a supervised or non-supervised mode. The non-supervised mode uses empirical Bayes networks (implemented through "ComBat.R", [1]) to adjust for batch effects between the individual datasets. In

the supervised mode the user assigns additional covariates next to the batch assignment, such as ”treated” and ”untreated”, or ”non-differentiated” and ”differentiated”. Note that this information has to be valid, as it impacts on the results you will get.

Last but not least it is possible to use the package to integrate data without batch effect removal, so that other, user-defined, methods of batch effect removal can be employed.

The combined data is presented as a regular Bioconductor ”ExpressionSet” object, which permits using all of R/Bioconductor’s power on the dataset as a whole.

To load the package type:

```
> library(virtualArray)

To cite package 'virtualArray' in publications use:

  Andreas Heider and RÃijdiger Alt (2013). virtualArray: a
  R/bioconductor package to merge raw data from different
  microarray platforms. R package version 1.4.1.

A BibTeX entry for LaTeX users is

  @Article{,
    title = {virtualArray: a R/bioconductor package to merge raw data from different m
    author = {{Andreas Heider} and {RÃijdiger Alt}},
    year = {2013},
    note = {R package version 1.4.1},
    journal = {BMC Bioinformatics},
    volume = {14},
    pages = {75},
  }
```

2 More in depth step by step explanations

Now that you read the introduction I will explain in more detail the different steps that are made during processing and finally combining the data to the new virtual array.

1. The raw data of each chip/platform or simply batch are to be read in to form an ExpressionSet in Bioconductor. This is to be done by means of other packages e.g. ”affy”, ”lumi” or ”limma”. Depending on the chip or package used, you may have to fill or fix the ”annotation” slot of the ExpressionSet to contain available Bioconductor annotation packages without the ”*.db” extension. This is particularly important when pulling data from NCBI GEO or EBI ArrayExpress.

2. Each batch, even if it is based on the same platform, may have been scanned in using different hardware or different modes of usage. Because of this, we need to transform each dataset to common scale (log2, log10 or linear) and resolution (12, 14 , 16 or 20 bit). Again, we can do this using standard R functions on the ”exprs” slot of the ExpressionSets.

3. When we want to combine data from different platforms we cannot use manufacturer identifiers like 1000_s_at or ILM_123456 to find matching pairs. Indeed we have to annotate each dataset with additional identifiers. When starting the processing using e.g. ”virtualArrayExpressionSets()” we define which additional identifier to pull from the annotation package. The default is to use gene symbols (named ”SYMBOL” in the annotation packages). However, it can be anything present in the annotation packages that gives a 1:1 mapping of identifiers. This will be fixed in future versions of the package.

4. Before attempting to match common identifiers we need to collapse rows that target the same identifier or gene in the default case. This is done by either ”median” (the default) or a user supplied function. This results in a reduced expression matrix.

5. Now we proceed with matching common identifiers. A new expression matrix is built, that just includes rows for identifiers present in all datasets.

6. virtualArray constructs a new ExpressionSet object with the just built expression matrix and a ”pData” that contains relations between batches and samples.

7. The newly generated ExpressionSet can now either be returned without further modifications or directly subjected to batch effect removal using empirical Bayes methods or another method of choice. Please see the documentation of the ”virtualArray.ExpressionSets” function for details. This can be decided by the user with the logical or character vector ”removeBatchEffects”.

   Note, however, that the contents of the resulting ExpressionSet is not, and actually can not be, a simple concatenation of the input expression matrices. On the one hand incompatible probes/probesets are excluded during the process. On the other hand expression values targetting the same identifier (e.g. gene) are collapsed by the function (e.g. ”median”) defined in the first place.

   The option to not remove the batch effect has been implemented, so you can actually see the impact of it in the combined data. In other words, you could not create a figure like in section 3.3. (first figure) in an easy way without using this package. Furthermore this option can be used to pass the non-corrected ExpressionSet on to other batch effect removal methods.

3 A short example with real world data

I will now go through a short example using the ”virtualArrayExpressionSets” approach.

For this we will have to pull some data from the NCBI GEO database. I selected two induced pluripotent stem cell (iPSCs) datasets. The first one includes human embryonic stem cells (ESCs), fibroblasts and iPSCs [2]. The second one includes fibroblasts and iPSCs derived from them [3]. The task in this example is to combine and compare data from highly similar cell types that were generated on different platforms: the Agilent G4112A and the Affymetrix HG-U133Plus2 respectively.

3.1 Loading and preparing the data

We use the getGEO function from the GEOquery package to retrieve the desired GEO series.

```
> library("GEOquery")
> GSE23402 <- getGEO("GSE23402",GSEMatrix=T,AnnotGPL=FALSE)
> GSE26428 <- getGEO("GSE26428",GSEMatrix=T,AnnotGPL=FALSE)
```

We select a subset of the data, so the example will run faster.

```
> GSE23402 <- GSE23402[[1]][,1:24]
> GSE26428 <- GSE26428[[1]]
```

Agilent and Affimetrix use different raw data formats. Therefore it is absolutely essential to check if the raw data need to be transformed initially. Let's have a look at the data with the simple summary function.

```
> summary(exprs(GSE23402)[,1:3])
```

```
   GSM574058           GSM574059           GSM574060
 Min.   :   10.06   Min.   :   10.06   Min.   :   10.06
 1st Qu.:   22.92   1st Qu.:   22.92   1st Qu.:   22.92
 Median :   59.03   Median :   59.03   Median :   59.03
 Mean   :  510.00   Mean   :  510.00   Mean   :  510.00
 3rd Qu.:  282.19   3rd Qu.:  282.19   3rd Qu.:  282.19
 Max.   :28094.15   Max.   :28094.15   Max.   :28094.15
```

```
> summary(exprs(GSE26428))
```

```
   GSM648497         GSM648498         GSM648499
 Min.   : 1.054   Min.   : 1.054   Min.   : 1.054
 1st Qu.: 3.080   1st Qu.: 3.080   1st Qu.: 3.080
 Median : 7.062   Median : 7.062   Median : 7.062
 Mean   : 6.983   Mean   : 6.983   Mean   : 6.983
 3rd Qu.:10.233   3rd Qu.:10.233   3rd Qu.:10.233
 Max.   :18.578   Max.   :18.578   Max.   :18.578
```

We can see that GSE23402 data is not log-scaled, as we have values ranging high over 20000. On the other hand GSE26428 data is in log-scale at a resolution of 20 bit, because we have all values under 100. The maxima are above 16 and 17, but below 20 and 19. Now we cannot have 17 or 19 bit, but 20 bit. Also you can find information from Agilent, that the chip used here is scanned at 20 bit resolution.

To resolve this problem, we log2-transform the Affymetrix data, and transform the Agilent data from 20 bit to 16 bit resolution.

```
> exprs(GSE23402) <- log2(exprs(GSE23402))
> exprs(GSE26428) <- (exprs(GSE26428)/20*16)
```

Here are the results of our transformations. You can appreciate that both datasets now spread to the same data format: 16 bit log2-transformed.

```
> summary(exprs(GSE23402)[,1:4])
```

```
   GSM574058          GSM574059          GSM574060          GSM574061
 Min.   : 3.331   Min.   : 3.331   Min.   : 3.331   Min.   : 3.331
 1st Qu.: 4.519   1st Qu.: 4.519   1st Qu.: 4.519   1st Qu.: 4.519
 Median : 5.883   Median : 5.883   Median : 5.883   Median : 5.883
 Mean   : 6.491   Mean   : 6.491   Mean   : 6.491   Mean   : 6.491
 3rd Qu.: 8.140   3rd Qu.: 8.140   3rd Qu.: 8.140   3rd Qu.: 8.140
 Max.   :14.778   Max.   :14.778   Max.   :14.778   Max.   :14.778
```

```
> summary(exprs(GSE26428))
```

```
   GSM648497          GSM648498          GSM648499
 Min.   : 0.8433   Min.   : 0.8433   Min.   : 0.8433
 1st Qu.: 2.4636   1st Qu.: 2.4636   1st Qu.: 2.4636
 Median : 5.6495   Median : 5.6495   Median : 5.6495
 Mean   : 5.5863   Mean   : 5.5863   Mean   : 5.5863
 3rd Qu.: 8.1861   3rd Qu.: 8.1861   3rd Qu.: 8.1861
 Max.   :14.8623   Max.   :14.8623   Max.   :14.8623
```

We next need to set the correct Bioconductor annotations. UPDATE: New virtualArray version automatically translates known GPL Ids to Bioc annotations via the "GPLs" list (see documentation).

```
> annotation(GSE23402) <- "hgu133plus2"
> annotation(GSE26428) <- "hgug4112a"
```

Now we create an empty object to hold our virtual arrays

```
> my_virtualArrays <- NULL
```

We have generated two ExpressionSets, which have their expression data in the same ”space”, and also have the correct annotation packages attached to them. We have also created an empty object to hold the ExpressionSets being generated. This is necessary, because we will create 2 of them and the function we call next, scans the current environment for ExpressionSets to combine them. So if we stored it in the current environment, we would end up adding our newly generated virtual array to the input data in the second run.

3.2 Building the virtual array

In the next step, we will compile the new ExpressionSet.

```
> my_virtualArrays$iPSC_hESC_noBatchEffect <- virtualArrayExpressionSets()
```

```
The downloaded source packages are in
        âĂŸ/tmp/RtmpfqhcLH/downloaded_packagesâĂŹ
Reading Sample Information File
Reading Expression Data File
Found 2 batches
Found 0 covariate(s)
Standardizing Data across genes
Fitting L/S model and finding priors
Finding parametric adjustments
Adjusting the Data
          full vs.GSE23402 vs.GSE26428 vs.result
GSE23402 19944       19944       17707     17707
GSE26428 18985       17707       18985     17707
result   17707       17707       17707     17707
```

We will now compile a second ExpressionSet WITHOUT REMOVING BATCH EFFECTS.

```
> my_virtualArrays$iPSC_hESC_withBatchEffect <- virtualArrayExpressionSets(removeBat
```

```
          full vs.GSE23402 vs.GSE26428 vs.result
GSE23402 19944       19944       17707     17707
GSE26428 18985       17707       18985     17707
result   17707       17707       17707     17707
```

This step just adds some sensible phenoData, including colors(!).

```
> pData(my_virtualArrays$iPSC_hESC_noBatchEffect)[5] <-
+         c(as.character(pData(GSE23402)[,8]),as.character(pData(GSE26428)[,1]))
> pData(my_virtualArrays$iPSC_hESC_noBatchEffect)[6] <-
+         c(rep("red",24),rep("blue1",3))
```

The virtualArray package is now completely executed. At this stage one could proceed with your intended analysis of the first ExpressionSet, or apply an alternative batch effect removal method using the second ExpressionSet generated without batch effect removal.

Note that we used some defaults of the function that are good to know about:

We used gene symbols (identifiers="SYMBOL") to annotate each expression matrix and we selected the median (collapse_fun=median) of rows targetting the same gene symbol (that is e.g. Affymetrix probe sets targeting the same gene). This results in the removal of redundancies and the reduction of the number of rows in each expression matrix (see the output above).

Furthermore, the package removes gaps by selecting only those entries that are present in all datasets. This is necessary, because all platforms detect slightly different fractions of the genome, but not the whole genome. Hence, the number of rows is further reduced.

3.3 A glimpse at the results

To have a first look at the data, we create distance matrices and perform a hierarchical clustering. Distances between observations are calculated using euclidian distances.

```
> dist_iPSC_hESC_noBatchEffect <-
+         dist(t(exprs(my_virtualArrays$iPSC_hESC_noBatchEffect)),
+         method="euclidian")
```

```
> dist_iPSC_hESC_withBatchEffect <-
+         dist(t(exprs(my_virtualArrays$iPSC_hESC_withBatchEffect)),
+         method="euclidian")
```

Trees are formed using average distance between clusters

```
> hc_iPSC_hESC_noBatchEffect <-
+         hclust(dist_iPSC_hESC_noBatchEffect, method="average")
> hc_iPSC_hESC_noBatchEffect$call <- NULL
```

```
> hc_iPSC_hESC_withBatchEffect <-
+         hclust(dist_iPSC_hESC_withBatchEffect, method="average")
> hc_iPSC_hESC_withBatchEffect$call <- NULL
```

We will plot the hclust in color, because the naming itself isn't very clear

```
> virtualArrayHclust(hc_iPSC_hESC_withBatchEffect,
+         lab.col=pData(my_virtualArrays$iPSC_hESC_noBatchEffect)[,6],
+         lab=pData(my_virtualArrays$iPSC_hESC_noBatchEffect)[,5],
+         main="batch effect NOT removed",cex=0.7,
+         xlab="sample names")
```

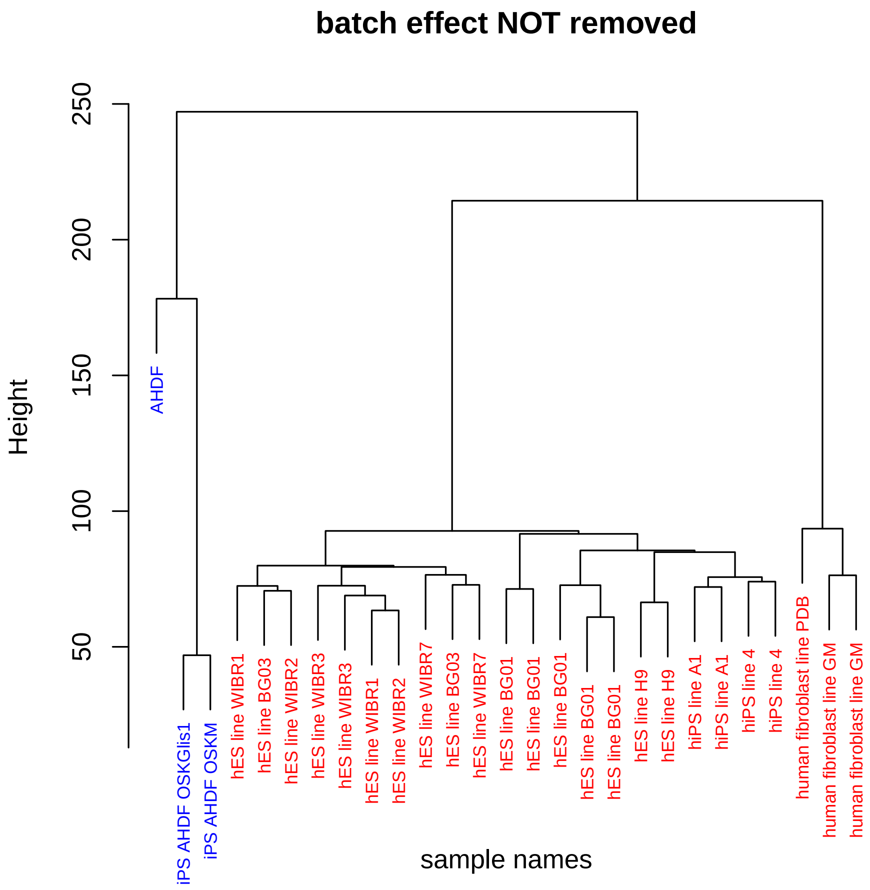


```
> virtualArrayHclust(hc_iPSC_hESC_noBatchEffect,
+         lab.col=pData(my_virtualArrays$iPSC_hESC_noBatchEffect)[,6],
+         lab=pData(my_virtualArrays$iPSC_hESC_noBatchEffect)[,5],
+         main="batch effect removed",cex=0.7,
+         xlab="sample names")
```

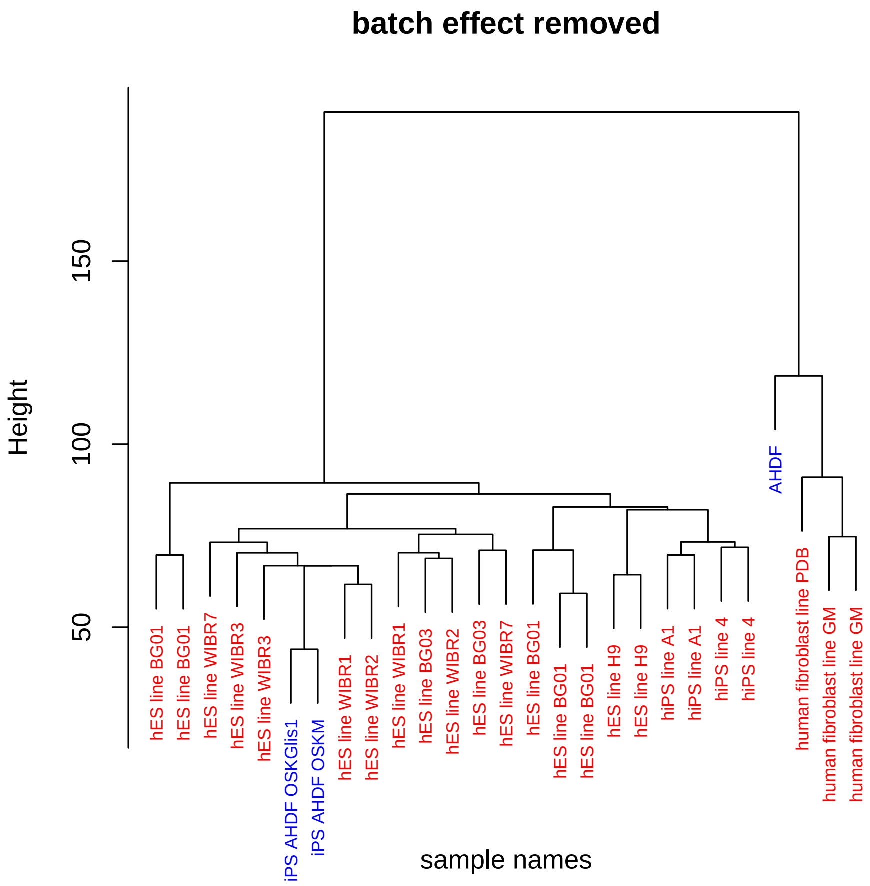

We colored all samples from Guenther et al. in red and all sampels from Maekama et al. in blue. the first image shows, that there are indeed significant batch effects between the two experiments, as fibroblasts and iPS cells cluster according to the platform they were generated on, rather than according to their biological similarity. After batch effect removal, we see that the non-supervised mode yields a significant improvement. The terminally differentiated fibroblasts now cluster together, whereas the pluripotent iPS cells cluster together with pluripotent ESC lines, irrespective of the origin of the data.

3.4 A final run in supervised mode

In order to get the supervised mode to run, we need to edit the "sample_info.txt" file, that is written to the current working directory on the fly. We will modify the 4th column to look like in table 1. UPDATE: The new virtualArray version can use a common column in the pData slots of the supplied ExpressionSets, please see documentation for details.

Note, that we have thus chosen to order the samples into 2 groups in addition to the 2 batches. This tells the algorithm that we expect the biological variances to be greatest

Table 1: sample_info.txt

	Array.name	*Sample.name*	*Batch*	*Covariate.1*
1	GSM574058	GSM574058	GSE23402	fibroblast
2	GSM574059	GSM574059	GSE23402	fibroblast
3	GSM574060	GSM574060	GSE23402	fibroblast
4	GSM574061	GSM574061	GSE23402	ESC_or_iPSC
5	GSM574062	GSM574062	GSE23402	ESC_or_iPSC
6	GSM574063	GSM574063	GSE23402	ESC_or_iPSC
7	GSM574064	GSM574064	GSE23402	ESC_or_iPSC
8	GSM574065	GSM574065	GSE23402	ESC_or_iPSC
9	GSM574066	GSM574066	GSE23402	ESC_or_iPSC
10	GSM574067	GSM574067	GSE23402	ESC_or_iPSC
11	GSM574068	GSM574068	GSE23402	ESC_or_iPSC
12	GSM574069	GSM574069	GSE23402	ESC_or_iPSC
13	GSM574070	GSM574070	GSE23402	ESC_or_iPSC
14	GSM574071	GSM574071	GSE23402	ESC_or_iPSC
15	GSM574072	GSM574072	GSE23402	ESC_or_iPSC
16	GSM574073	GSM574073	GSE23402	ESC_or_iPSC
17	GSM574074	GSM574074	GSE23402	ESC_or_iPSC
18	GSM574075	GSM574075	GSE23402	ESC_or_iPSC
19	GSM574076	GSM574076	GSE23402	ESC_or_iPSC
20	GSM574077	GSM574077	GSE23402	ESC_or_iPSC
21	GSM574078	GSM574078	GSE23402	ESC_or_iPSC
22	GSM574079	GSM574079	GSE23402	ESC_or_iPSC
23	GSM574080	GSM574080	GSE23402	ESC_or_iPSC
24	GSM574081	GSM574081	GSE23402	ESC_or_iPSC
25	GSM648497	GSM648497	GSE26428	ESC_or_iPSC
26	GSM648498	GSM648498	GSE26428	ESC_or_iPSC
27	GSM648499	GSM648499	GSE26428	fibroblast

between these 2 groups. Now we run the package once again, but this time we need to modify the "sample_info.txt" file when prompted. After editing please select "y" to run in supervised mode.

```
> my_virtualArrays$iPSC_hESC_supervised <- virtualArrayExpressionSets(sampleinfo="cr

Reading Sample Information File
Reading Expression Data File
Found 2 batches
Found 0 covariate(s)
Standardizing Data across genes
Fitting L/S model and finding priors
Finding parametric adjustments
Adjusting the Data
          full vs.GSE23402 vs.GSE26428 vs.result
GSE23402 19944       19944       17707     17707
GSE26428 18985       17707       18985     17707
result   17707       17707       17707     17707

> dist_iPSC_hESC_supervised <-
+         dist(t(exprs(my_virtualArrays$iPSC_hESC_supervised)),
+         method="euclidian")

> hc_iPSC_hESC_supervised <<-
+         hclust(dist_iPSC_hESC_supervised, method="average")
> hc_iPSC_hESC_supervised$call <- NULL
```

Again we will plot the hclust in color, because the nomenclature alone is not sufficient.

```
> virtualArrayHclust(hc_iPSC_hESC_supervised,
+         lab.col=pData(my_virtualArrays$iPSC_hESC_noBatchEffect)[,6],
+         lab=pData(my_virtualArrays$iPSC_hESC_noBatchEffect)[,5],
+         main="batch effect removed - supervised mode",cex=0.7,
+         xlab="sample names")
```

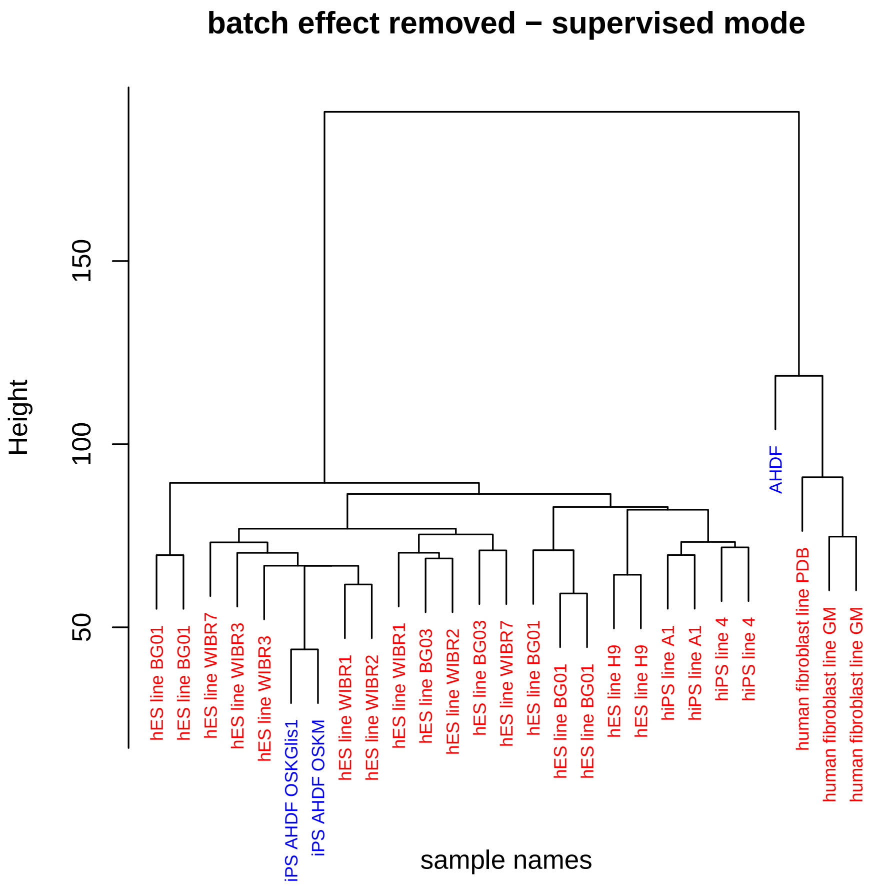

To get a final view of the data we perform a principle component analysis. Coloring is based on batches.

```
> pca_supervised <- prcomp(t(exprs(my_virtualArrays$iPSC_hESC_supervised)))
> plot(pca_supervised$x, pch=19, cex=2, col=c(rep("red",24),rep("blue",3),pch=17))
> legend("topleft",c("GSE23402","GSE26428"),col=c("red","blue"),pch=19,cex=1)
```

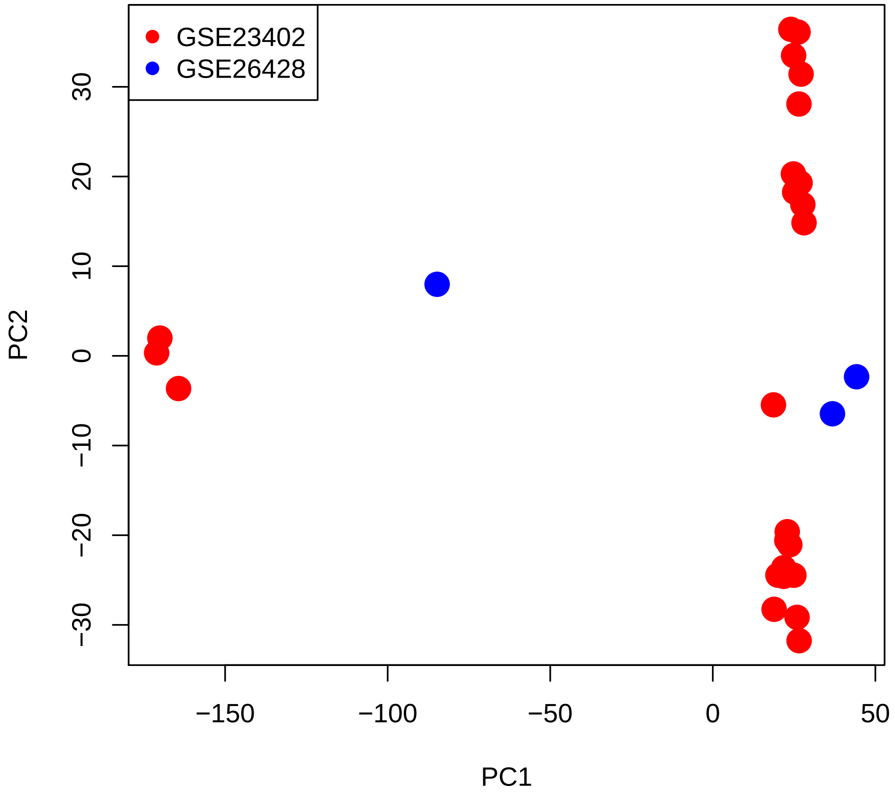

You can see that especially the fibroblasts cluster together more closely now. On the other hand each sample is on the same relative position, as when running in non-supervised mode. This indicates, that the batch effects have been further reduced, without losing biological information.

This example shows, how "virtualArray" can be used to integrate samples from different labs generated on different platforms into one virtual array. Similarly, published datasets from two or more clinical studies could be combined into one large raw data based meta-analysis, e.g. to investigate transcriptional signatures of malignant tissues. Therefore, I hope this tool will be valuable for basic as well as clinical researchers.

Now have fun using it!

4 Literature

1. **Li C, Rabinovic A.** Adjusting batch effects in microarray expression data using empirical Bayes methods. **Biostatistics** 2007;8:118-127.

2. **Guenther et al.** Chromatin structure and gene expression programs of human

embryonic and induced pluripotent stem cells. **Cell Stem Cell** 2010;7(2);249-57.

3. **Maekawa et al.** Direct reprogramming of somatic cells is promoted by maternal transcription factor Glis1. **Nature** 2011;474(7350);225-9.
